# Structural basis of RGD-hirudin binding to thrombin: Tyr_3_ and five C-terminal residues are crucial for inhibiting thrombin activity

**DOI:** 10.1186/s12900-014-0026-9

**Published:** 2014-12-20

**Authors:** Yinong Huang, Yanling Zhang, Bing Zhao, Qiping Xu, Xiushi Zhou, Houyan Song, Min Yu, Wei Mo

**Affiliations:** Key Laboratory of Metabolism and Molecular Medicine, ministry of education, Fudan University, Shanghai, China; The Department of Biochemistry and Molecular Biology, School of Basic Medical Sciences, Fudan University, Shanghai, China; Collaborative Innovation Center for Biotherapy, Sichuan University, Chengdu, China

**Keywords:** Recombinant RGD-hirudin, Thrombin, Molecular simulation, Surface plasmon resonance, Affinity constants

## Abstract

**Background:**

Hirudin is an anti-coagulation protein produced by the salivary glands of the medicinal leech Hirudomedicinalis. It is a powerful and specific thrombin inhibitor. The novel recombinant hirudin, RGD-hirudin, which contains an RGD motif, competitively inhibits the binding of fibrinogen to GPIIb/IIIa on platelets, thus inhibiting platelet aggregation while maintaining its anticoagulant activity.

**Results:**

Recombinant RGD-hirudin and six mutant variants (Y3A, S50A, Q53A, D55A, E57A and I59A), designed based on molecular simulations, were expressed in Pichia pastoris. The proteins were refolded and purified to homogeneity as monomers by gel filtration and anion exchange chromatography. The anti-thrombin activity of the six mutants and RGD-hirudin was tested. Further, we evaluated the binding of the mutant variants and RGD-hirudin to thrombin using BIAcore surface plasmon resonance analysis (SPR). Kinetics and affinity constants showed that the K_D_ values of all six mutant proteins were higher than that of RGD-hirudin.

**Conclusions:**

These findings contribute to a novel understanding of the interaction between RGD-hirudin and thrombin.

## Background

A highly specific thrombin inhibitor derived from the leech (*Hirudomedicinalis hirudin*) has come into use over the past decade in *in vitro* blood research. It blocks the thrombin-mediated conversion of fibrinogen to fibrin during clot formation, but unlike heparin, it is a direct thrombin inhibitor (DTI) [1] that is not inactivated by platelet factor 4 (PF4) [2,3].

A previous study has shown that the inhibitor is a small peptide (65 amino acids, 7 kDa) that binds to active thrombin and irreversibly inactivates it [4]. It has also been thoroughly characterized in several laboratories by biochemical and biophysical means, including determination of nuclear magnetic resonance (NMR) structures in solution of both natural and recombinant variants [5-7]. These investigations revealed that hirudin comprises an N-terminal globular domain (residues Ile_1_-Ile_49_) stabilized by three disulfide bonds with [1-5,4-6] connectivity, which spontaneously folds in solution [8]. This compact domain is extended on the C-terminus by a short acidic tail that lacks cysteine residues and is essentially disordered in solution [9]. Structural studies conducted on hirudin in the free [5-7,10] and thrombin-bound state [11,12] indicate that both the N-terminus (Tyr_3_, Asp_5_) [11,13] and the C-terminus play an important role in the interaction with thrombin. Notably, the long, extended conformation of the C-terminus interacts with a multitude of residues on the surface of thrombin.

The novel recombinant RGD-hirudin, which contains an Arg-Gly-Asp (RGD) adhesion site recognition sequence, is a bi-functional molecule based on the structure of wild-type hirudin variant 2 [14]. In the recombinant version, several amino acid residues in the C-terminus have been replaced by negatively charged residues (Asp_62_ and Asp_65_). Asp_53_ was mutated to Gln_53_, Glu_58_ was mutated to Pro_58_, and Glu_66_ was added. These changes improve the hydrophobicity of the protein and allow the recombinant RGD-hirudin to interact more effectively with the fibrinogen recognition exosite of thrombin, resulting in a specific activity of 12,000 ATU/mg [15]. Given these changes, we hypothesized that the interaction between RGD-hirudin and thrombin would be similar to that between wild-type hirudin and thrombin. To test this, we expressed and purified RGD-hirdudin and six mutant variants in *Pichia pastoris*. The design of the six mutations was based on molecular simulations. The Titration Testing Method and BIAcore® surface plasmon resonance analysis (SPR) were used to test their thrombin inhibition activity. Anti-platelet aggregation activity was tested by classic turbidity assays.

## Methods

### Materials

Accelrys Discovery Studio (DS, version 3.1) was used for homology modeling (MODELER) [16] and docking simulation (ZDOCK) [17,18]. *Pichia pastoris* cells carrying the RGD-hirudin gene (Mut^+^) and pPIC9k-RGD-hirudin plasmid were stored in our lab. Briefly, the RGD-hirudin gene was synthesized in the Key Laboratory of Molecular Medicine at Fudan University. cDNA encoding RGD-hirudin was cloned into the plasmid pPIC9K, and this expression vector was transformed into *Pichia pastoris* GS115. Vector integration into the *Pichia pastoris* chromosome was confirmed by PCR [14,19]. DNA primers were synthesized by Sangon Biotech (Shanghai) Co., Ltd. The Site-Directed Mutagenesis Kit was purchased from SBS Genetech Co., Ltd. Yeast nitrogen base was obtained from Sigma Aldrich Co., Ltd. Blood plasma was obtained from the Shanghai Blood Center. Sephacryl S-100 HR, Sephadex-G50, and Q-Sepharose-FF were purchased from GE Healthcare Co., Ltd. The Biacore T100 instrument and research grade CM5 chips were purchased from Biacore (GE Healthcare) Co., Ltd. Other reagents were of analytical purity.

### Homology modeling

The amino acid sequence of RGD-hirudin was obtained in our lab. The NCBI protein BLAST program was used to search the Protein Data Bank (PDB) and was used to select a template structure for RGD-hirudin homology modeling. From the selected templates, a three-dimensional model of RGD-hirudin was obtained by homology modeling using the software package Discovery Studio 3.1. Constructed models were refined by performing an optimized geometry calculation of the mechanics using augmented CHARMM in Discovery Studio 3.1. The quality of refined models was assessed on the basis of both geometry and energy. The stereo-chemical properties of the models were investigated with a Ramachandran plot using PROCHECK [20].

### Protein-protein docking

To assess the interaction between RGD-hirudin and thrombin, docking the modeled structure of RGD-hirudin with the crystal structure of thrombin (PDB ID: 4HTC) chain H&L was performed. ZDOCK tested the different docking sites by moving the ligand around the receptor. To obtain more accurate predictions, we specified the angular step size for the rotational sampling of the ligand orientations as 6, and the shape complementarity, desolvation and electrostatic energy terms were used in the initial-stage ranking of the docked protein poses. The docking parameters are set as follow: Input Receptor Protein: 4HTC: Thrombin, Input Ligand Protein: RGD-Hirudin, Angular Step Size: 6, ZRank: True, Clustering: Top poses: 2000, RMSD Cutoff: 6, Interface Cutoff: 9, Maximum Number of Clusters: 60.

### Mutation Energy Calculation (binding affinity)

The Mutation Energy Calculation was used to evaluate the effect of single-point mutations on the binding affinity in the RGD-hirudin-thrombin docking complex. This protocol performs amino acid scanning mutagenesis on a set of residues (selected according to the docking results) by mutating each to alanine. The energy effect on the binding affinity of each mutation (Mutation Energy) was calculated as the difference in the binding free energy between the mutant and wild-type proteins. The binding free energy is defined as the difference in the free energy between the complexed and unbound state. All interaction energy terms were calculated by CHARM using a Generalized Born implicit solvent model; these terms contained empirically scaled contributions of van der Waals and electrostatic interactions and a non-polar solvation energy term. The mutation energy function contained also a side chain entropy term. The temperature was set at 37°C. This process was also implemented with the software package Discovery Studio 3.1.

### Cloning, expression, and purification of RGD-hirudin and the mutant variants

For preparation of six RGD-hirudin mutants, a Site-Directed Mutagenesis Kit was using two complementary primers which were described as follows (Table 1) and the pPIC9k-RGD-hirudin plasmid as a template. The procedure consisted of 18 polymerase chain reaction cycles as specified by the manufacturer’s manual using Pfu polymerase. The mutated construct was then digested with the restriction enzyme DpnI, which is specific to methylated and hemi-methylated DNA, to digest the template and select mutation-containing newly-synthesized DNA. DNA sequencing confirmed that all six mutant plasmids were correct.

**Table 1:** **Primers used for the generation of RGD-hirudin mutants**

**Primer**	Primer sequence*
Y3A-5′	GAGAAAAGAGTTGTT**GCT**ACTGACTGCACTGAA
Y3A-3′	TTCAGTGCAGTCAGT**AGC**AACAACTCTTTTCTC
S50A-5′	ACCCCGAAACCGCAG**GCT**CACAACCAGGGTGAC
S50A-3′	GTCACCCTGGTTGTG**AGC**CTGCGGTTTCGGGGT
Q53A-5′	CCGCAGTCCCACAAC**GCT**GGTGACTTCGAACCG
Q53A-3′	CGGTTCGAAGTCACC**AGC**GTTGTGGGACTGCGG
D55A-5′	TCCCACAACCAGGGT**GCT**TTCGAACCGATCCCG
D55A-3′	CGGGATCGGTTCGAA**AGC**ACCCTGGTTGTGGGA
E57A-5′	AACCAGGGTGACTTC**GCT**CCGATCCCGGAAGAC
E57A-3′	GTCTTCCGGGATCGG**AGC**GAAGTCACCCTGGTT
I59A-5′	GGTGACTTCGAACCG**GCT**CCGGAAGACGCTTAC
I59A-3′	GTAAGCGTCTTCCGG**AGC**CGGTTCGAAGTCACC

*Sense primer-5′; anti-sense primer-3′; all mutations are in bold letters.

Wild type and mutant variants of RGD-hirudin were expressed in *Pichia pastoris,* then purified by gel filtration and anion exchange chromatography as previously described [14,21]. Briefly, a single colony was used to inoculate 5 mL of YPD in a 50 mL flask, which was incubated for 14 h at 30°C at 220 rpm. This culture was used to inoculate 200 mL of sterile BMGY. Cultures were incubated at 30°C and 220 rpm for 30 h to an OD_600_ of 5.8. Cells were harvested by centrifugation at 3000 rpm for 30 min, washed with 100 mM potassium phosphate (pH 6.0), and carefully resuspended in 200 mL sterile BMM. Samples were incubated at 30°C for another 72 h, with pulses of 0.5% (v/v) methanol added every 12 h. The culture was centrifuged and the supernatant was loaded onto a Sephacryl-S100 column (7.5 cm × 80 cm), pre-equilibrated with 20mMol/L phosphate buffer (PB, pH 7.4). A sample of 400 mL was eluted from the gel filtration column and loaded onto a Q-Sepharose FF column (2.6 cm × 20 cm), also pre-equilibrated with 20mMol/L PB (pH 7.4). The column was washed with 20mMol/L PB (pH 7.4), followed by a single linear gradient of 0–1.0Mol/L NaCl-PB buffer. RGD-hirudin was eluted at 0.25Mol/L NaCl-PB. The sample that showed anti-thrombin activity was collected and desalted with a Sephadex-G50 column (1.6 cm × 20 cm). Loading sample volumes were standardized to 5 mL. Protein concentration was measured with the Bradford assay. The desalted samples were lyophilized and stored at −80°C.

### Protein identification

Protein samples (wild type and mutant variants of RGD-hirudin) were analyzed by 15% SDS-PAGE. The anti-thrombin activity of RGD-hirudin and the mutant variants was tested by the Titration Testing Method, according to Markwardt [22]. Briefly, 200 mL of fresh plasma was added to a 1.5 mL tube. A 5 mL sample of RGD-hirudin was added to the plasma and mixed by vortexing. Thrombin (5 mL of 100 NIH units) was added and allowed to stand for 1 min at 37°C. If the plasma did not clot, the RGD-hirudin contained 100 units of anti-thrombin activity. Thus, consumption of 1 NIH unit of thrombin is equivalent to 1 unit of anti-thrombin activity. A classic turbidity assay was used to measure the anti-platelet aggregation activity of RGD-hirudin and the mutant variants [15]. Aggregation of rabit platelets in response to antagonists was analyzed using lumi-aggregometer (Model 400VS, Chrono-Log, Haverston, PA, USA).

### Surface plasmon resonance interaction analysis (BIAcore)

Thrombin was diluted in 5mMol/L sodium acetate (pH 5.0), and 2000 response units were immobilized via amine coupling to CM5 sensor chip flow chambers (GE Healthcare). Briefly, proteins were mixed with equal volumes of freshly prepared 100mMol/L N-hydroxysuccinimide and 400mMol/L N-ethyl-N’-(dimethylaminopropyl) carbodiimide, and capping of unreacted carboxymethyl sites was achieved by injection of 1.0Mol/L ethanolamine, pH 7.0. A flow chamber was subjected to the immobilization protocol. RGD-hirudin and the six mutant variants were sequentially diluted in running buffer (10mMol/L HEPES, 150mMol/L NaCl, 0.005% Surfactant P20 (BIAcore), pH 7.5) and injected over the surfaces at different concentrations (RDG-hirudin: 0-50nMol/L; Y3A: 0-10mMol/L; S50A: 0-50nMol/L; Q53A: 0-5μMol/L; D55A: 0-10μMol/L; E57A: 0-10μMol/L; I59A: 0-50μMol/L) at 30 μL/min.

Binding was monitored with a BIAcore T100 instrument. Between experiments, the surfaces were strictly regenerated with multiple pulses of 2Mol/L NaCl and 1.5Mol/L glycine-HCl, pH 2.5, followed by an extensive wash procedure with running buffer.

After x- and y-axis normalization of the data obtained, the blank bulk refraction curves from the control flow chamber of each injected concentration were subtracted. Binding curves were displayed, and the association (K_a_) and dissociation (K_d_) rate constants were determined using the BIAevaluation 4.1 software and its equation for 1:1 Langmuir binding. From these values, affinities (K_D_) were calculated.

## Results

### Molecular modeling of RGD-hirudin

**Figure 1:**
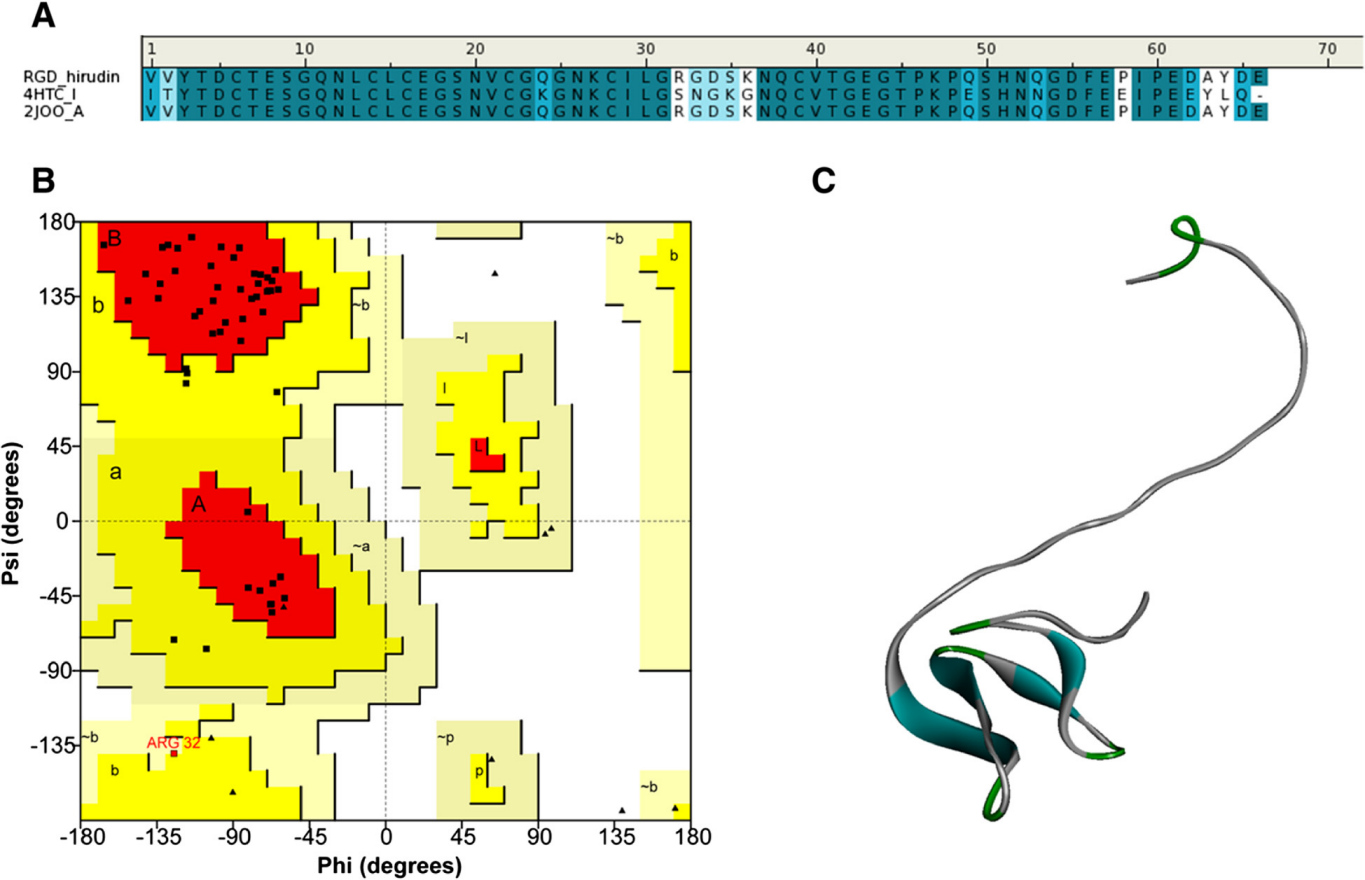
**Molecule simulation of RGD-hirudin.** **(A)** Amino acid sequence alignment of RGD-hirudin, chain I of hirudin variant 2 from the hirudin-thrombin complex (PDB ID: 4HTC) and the NMR solution structure of chain A of recombinant RGD-hirudin (PDB ID: 2JOO). **(B)** Ramachandran plot of the selected RGD-hirudin model. The distribution of the RGD-hirudin residues (black dots) are shown in color: most favorable (red), additional allowed (yellow), generously allowed (light yellow), and disallowed (white) regions. **(C)** Homology modeling of the RGD-hirudin structure.

RGD-hirudin was molecularly modeled, based on the crystal structure of hirudin variant 2 from chain I of the hirudin-thrombin complex (PDB ID: 4HTC) [23] and the NMR solution structure of chain A of recombinant RGD-hirudin (PDB ID: 2JOO) [24]. Chain I shares 80% and chain A shares 100% sequence identity with RGD-hirudin. Residues 52–66 of the structure, which may play important roles in the interaction with thrombin, were absent. The sequence alignment is shown in Figure 1A. The three-dimensional structural model for RGD-hirudin was generated based on the 4HTC and 2JOO templates using the Discovery Studio 3.1 software.

Analysis of the Ramachandran plot of the refined model showed that 96.9% of the residues lie in the most favorable regions (Figure 1B), which indicates that the model is suitable for structural studies (Figure 1C).

### Thrombin docked with RGD-hirudin

**Figure 2:**
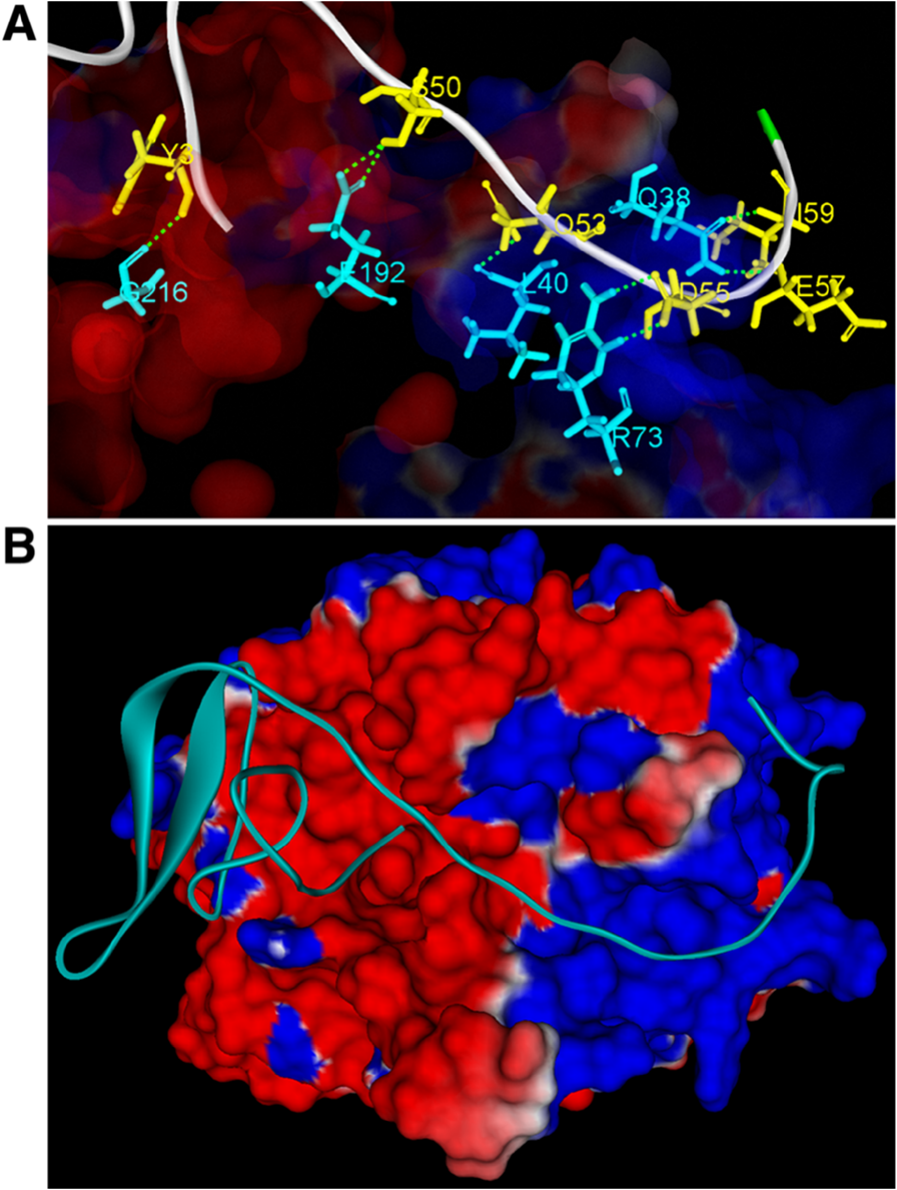
**Thrombin docked with RGD-hirudin.** Thrombin is represented by its solvent-accessible surface, while hirudin is shown as a solid ribbon. **(A)** The best docking complex of RGD-hirudin and thrombin (pose 24). **(B)** Close-up of the region of the RGD-hirudin functional site in complex with thrombin. Residues of RGD-hirudin are in yellow. Residues of thrombin are in blue. Hydrogen bond interactions are indicated with dashed green lines.

The docking simulation of RGD-hirudin and thrombin resulted in 54000 poses for analysis. The poses were clustered with the cluster root-mean-square deviation (RMSD) cutoff and the interface cutoff both set to 10 angstroms. For a pose to be included in a cluster, the RMSD cutoff was the maximal RMSD of the ligand interface from the cluster center, and the interface cutoff was the size of the interface region between receptor and ligand. Poses were then re-ranked with Van Der Waals, desolvation and electrostatics energy terms. The best docking poses were selected, based on a higher dock score combined with certain types of interactions between hirudin variant 2 and thrombin. We hypothesized that the interaction between RGD-hirudin and thrombin is similar to that of hirudin variant 2 and thrombin, and therefore favored this type of interaction. Finally, pose 24 was chosen as the best docking complex (Figure 2A). A close examination of the region in the functional site of RGD-hirudin when in complex with thrombin revealed that Tyr_3_ in the N-terminus of RGD-hirudin formed one hydrogen bond with Gly_210_ of thrombin. Further, in the C-terminus of RGD-hirudin, Ser_50_ formed two hydrogen bonds with Glu_192_ of thrombin, Gln_53_ formed one hydrogen bond with Leu_40_ of thrombin, Asp_55_ formed two hydrogen bonds with Arg_73_ of thrombin, and both Glu_57_ and Ile_59_ were hydrogen-bonded to Gln_38_ of thrombin (Figure 2B).

### Calculating the effect of single-point mutations on binding affinity

We performed amino acid scanning mutagenesis of RGD-hirudin-thrombin complexes, mutating to alanine each of six residues shown by the docking results to be important. We then calculated the differences in the free energy of binding between the wild-type and mutated structures. The results showed that the binding affinity of the six single-point mutations with thrombin were weaker (weighted mutation energy greater than zero) than that of wild-type RGD-hirudin with thrombin. The effect of each mutation was destabilizing (Table 2), which further confirmed the docking results and demonstrates that the six residues (Tyr_3_, Ser_50_, Gln_53_, Asp_55_, Glu_57_, Ile_59_) of RGD-hirudin might participate in the interaction with thrombin.

**Table 2:** Calculate the effect of single-point mutations on the binding affinity

**Residues**	**Mutation**	**VDW Term**	**Electrostatic Term**	**Entropy Term**	**Non-polar Term**	**Weighted Mutation Energy**	**Effect of Mutation**
Tyr3	Ala	6.58	-1.92	0.12	0	2.2	destabilizing
Ser50	Ala	6.68	0.41	-0.58	0	2.71	destabilizing
Gln53	Ala	8.99	-0.47	-2.02	0	2.16	destabilizing
Asp55	Ala	8.7	3.15	-0.94	0	4.56	destabilizing
Glu57	Ala	0.72	1.78	-0.13	0	1.02	destabilizing
Ile59	Ala	3.19	-0.4	-0.58	0	0.78	destabilizing

**VDW Term:** The van der Waals contribution to the binding energy differences.

**Electrostatic Term:** The electrostatic contribution to the binding energy difference between wild type and mutated structures.

**Entropy Term:** The side-chain entropy contribution to the binding energy differences.

**Non-polar Term:** The non-polar (surface tension) contribution to the binding energy differences.

**Weighted Mutation Energy:** The total free energy difference between the wild type and mutated structures. It is calculated as a weighted sum of the VDW, Electrostatic, Entropy and Non-polar terms.

### Purification and characterization of RGD-hirudin and mutant variants

**Figure 3:**
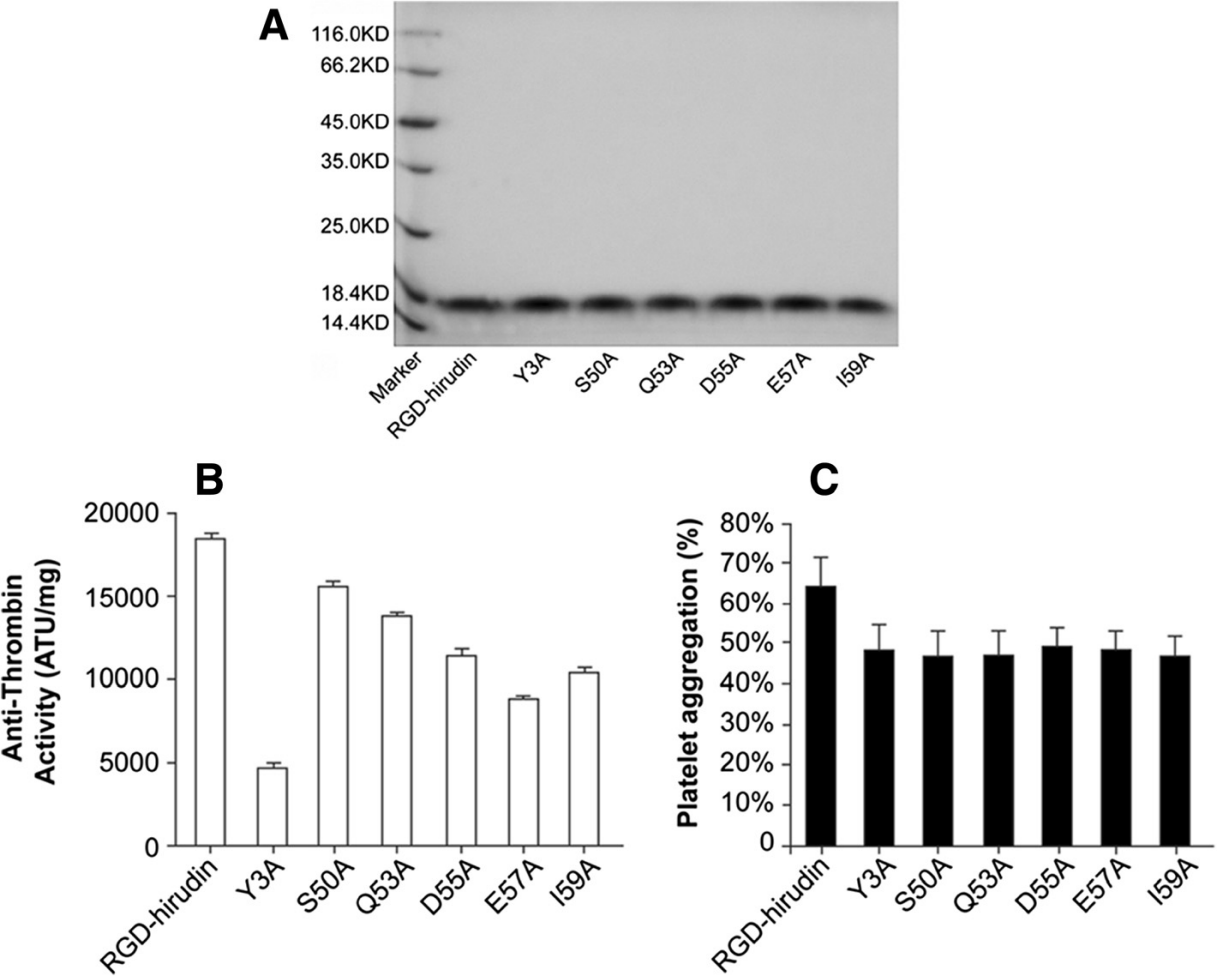
**Expression, purification and characteristic of RGD-hirudin and six mutant versions. (A)** Expression and purification of RGD-hirudin and six mutant versions were analyzed by 15% SDS-PAGE. Lane M: molecular mass standards (116 to 14.4 kDa) 20 μL (1 mg/mL) of each sample was loaded on the gel. **(B)** Anti-thrombin activity of purified RGD-hirudin and six mutant variants. **(C)** RGD-hirudin and the six mutants have similar effects on rabbit platelet aggregation induced by ADP. The experiments were performed five times with similar results. Error bars represent the standard deviation.

In this study, we focused on the residues of RGD-hirudin that are hydrogen-bonded to thrombin. These six residues (Tyr_3_, Ser_50_, Gln_53_, Asp_55_, Glu_57_, Ile_59_) were mutated to Ala, and the proteins were purified by gel filtration and anion exchange chromatography, as described in the Materials and Methods. The average yields of pure protein from 200 mL of fermentation culture were: RGD-hirudin, 46 mg; Y3A, 50 mg; S50A, 42 mg; Q53A, 47 mg; D55A, 40 mg; E57A, 38 mg; and I59A, 55 mg (Figure 3A).

The anti-thrombin specific activity of the purified RGD-hirudin was almost 18000 ATU/mg, while the mutated versions had activities reduced to different degrees: Y3A, 4000 Anti-thrombin Units (ATU)/mg; S50A, 16000 ATU/mg; Q53A, 14000 ATU/mg; D55A, 12000 ATU/mg; E57A, 11000 ATU/mg; and I59A, 9000 ATU/mg (Figure 3B). The dramatic decline in anti-thrombin specific activity of the Y3A mutation suggests that Tyr_3_ in RGD-hirudin might be a very important residue for its interaction with thrombin.

Anti-platelet aggregation activities of the proteins were determined by a classic turbidity assay. The results showed there was no significant difference between RGD-hirudin and the mutant variants (Figure 3C).

### SPR measurement of the interactions of RGD-hirudin and six mutant variants with thrombin

**Figure 4:**
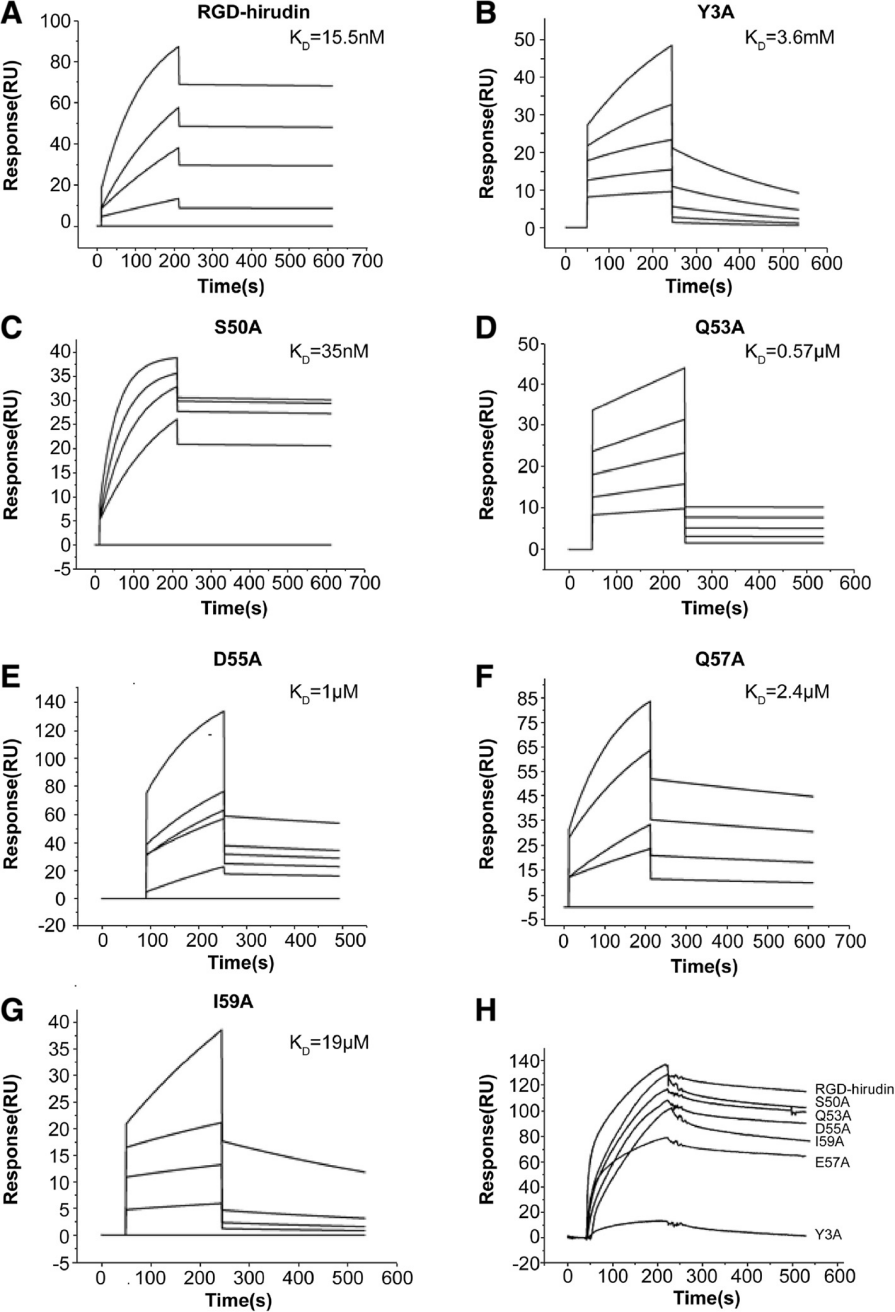
**SPR measurement of the interactions of RGD-hirudin and mutant variants with thrombin. (A-G)** K_D_ values. BIAcore analysis showed that the binding affinity of thrombin and wild-type RGD-hirudin was slightly stronger than those of the six mutants. **(H)** Affinity curves (with proteins at 100nM) demonstrated that the binding affinities of thrombin and the six mutants were weaker than that of RGD-hirudin.

To further analyze the binding properties of the six mutant variants of RGD-hirudin with thrombin and to establish the affinity constant of the binding, surface plasmon resonance analysis was performed. The binding activity of RGD-hirudin was compared with those of the six mutants (Y3A, S50A, Q53A, D55A, E57A and I59A) in a competitive non-radioactive binding assay using thrombin as a receptor. The K_D_ (affinity) was calculated from the determined K_a_ and K_d_ values (Table 3). The K_D_ for the interaction between RGD-hirudin and thrombin was 15.5nM (Figure 4A), whereas the K_D_ of thrombin binding for the mutant Y3A was 3.6 mM; S50A, 35nM; Q53A, 0.57 μM; D55A, 1 μM; E57A, 2.4 μM; and I59A, 19 μM (Figure 4B-G). BIAcore analysis thus confirmed that the binding affinity of thrombin and wild-type RGD-hirudin is slightly stronger than that of the S50A mutant, and much stronger than those of the other five variants, especially Y3A. Meanwhile, the affinity curves of RGD-hirudin and the six mutants (at the same molarity of 100nM) and thrombin demonstrated that the binding affinities for thrombin of all six mutant versions were weaker than that of RGD-hirudin (Figure 4H). In conclusion, the six residues (Tyr_3_, Ser_50_, Gln_53_, Asp_55_, Glu_57_ and Ile_59_) are important for binding to thrombin, as each mutant protein binds with lower affinity than wild-type RGD-hirudin.

**Table 3:** **Kinetics and affinity constants for RGD-hirudin and mutant variant binding to thrombin**

**Variant analyzed**	**Association rate k**_**a**_ **(M** ^**−1**^ **s** ^**−1**^ **)**	**Dissociation rate k**_**d**_ **(s** ^**−1**^ **)**	**Binding affinity K**_**D**_ **(M)**
RGD-hirudin	1.77 × 10^3^	2.73 × 10^−5^	1.55 × 10^−8^
Y3A	0.80	2.88 × 10^−3^	3.61 × 10^−3^
S50A	1.12 × 10^3^	3.94 × 10^−5^	3.50 × 10^−8^
Q53A	59.20	3.37 × 10^−5^	5.70 × 10^−7^
D55A	371.00	3.83 × 10^−4^	1.03 × 10^−6^
Q57A	155.00	3.74 × 10^−4^	2.41 × 10^−6^
I59A	73.00	1.39 × 10^−3^	1.90 × 10^−5^

## Discussion

Hirudin, an antithrombotic substance produced by the salivary glands of the medicinal leech (*Hirudomedicinalis*) [25,26], is the most potent and specific thrombin inhibitor currently known. It acts by binding directly via multiple sites to thrombin: the N-terminal globular domain binds near to the active site of thrombin, while the extended C-terminal segment, which is abundant in acidic residues and includes a sulfated tyrosine, has both ionic and hydrophobic interactions with the thrombin fibrinogen recognition exosite (FRE) [23].

In our laboratory, a new type of recombinant RGD-hirudin has been successfully cloned, expressed in the methylotrophic yeast *Pichia pastoris* and subsequently purified. It may be a more effective agent than wild-type hirudin for anti-coagulation and anti-thrombosis in post-anastomosis surgery. We propose that RGD-hirudin will be able to replace heparin or wild-type hirudin and reduce bleeding rates, due to its bi-functional action at a much lower dosage than wild-type hirudin [15]. However, RGD-hirudin needed to be modified to reduce the molecular weight to be suitable for advanced oral administration, rather than intravenous injection.

Although our lab has previously conducted a study on the structure of RGD-hirudin in solution (1–49) [24], the structure of the extended C-terminal domain (from Asn_52_ to Glu_66_), which is essential for the hirudin-thrombin interaction, had not yet been determined. The overall structure of RGD-hirudin (1–66) developed above used the crystal structure of chain I of hirudin variant 2 from the hirudin-thrombin complex (PDB ID: 4HTC) and the NMR solution structure of chain A of recombinant RGD-hirudin (PDB ID: 2JOO) as templates for docking with thrombin. Based on the docking model and the structural characteristics of the RGD-hirudin-thrombin complex, we find that the mechanism of interaction is consistent with that of native hirudin and thrombin. In this interaction, the globular N-terminal domains interact with the active site of thrombin, while the anionic C-terminal tails bind to exosite 1 on thrombin (the substrate-binding site) [27]. Six residues (Tyr_3_, Ser_50_, Gln_53_, Asp_55_, Glu_57_ and Ile_59_) of RGD-hirudin are hydrogen-bonded to thrombin, according to the results of simulations. To validate these residues as functional sites, mutant variants were constructed. Anti-thrombin activity assays and surface plasmon resonance analysis were also performed. Previous studies have indicated that the first three residues in the N-terminus of hirudin represent about 30% of the binding energy [28] and the acidic C-terminal segment accounts for about 32% of the binding energy at zero ionic strength [29]. Mutagenesis studies have shown that all the negatively charged residues of the C-terminal tail are relevant for complex formation [30-32]. Our results imply that RDG-hirudin’s N-terminus (Tyr_3_) and C-terminus (Ser_50_, Gln_53_, Asp_55_, Glu_57_ and Ile_59_) are both crucial for inhibiting the activity of thrombin, for binding near its active site and forming hydrogen bonds with exosite I.

In this study, we focused on hydrogen bonding interactions between RGD-hirudin and thrombin. However, hydrophobic interactions, Van der Waals interactions, and salt bridges might be also exist. For example, the phenyl ring of Phe_56_ engages in strong Van der Waals, edge-to-face interactions with thrombin Phe_34_, according to our docking complex (data not shown). Thus, other mechanisms of interacting and binding should be investigated in the future.

## Conclusions

In summary, The purpose of this study is that we found the binding site and active centre of RGD-Hirduin. In future, these results might be useful to provide a structural basis for the redesign of lower molecular weight antithrombin peptides which would be developed to the new oral drugs to fight thrombotic diseases.

## Abbreviations

RGD: Arginine-Glycine-Aspartic acid
SPR: Surface plasmon resonance analysis
DTI: Direct thrombin inhibitor
PF4: Platelet factor 4
NMR: Nuclear magnetic resonance
PB: Phosphate buffer
K_a_: Association rate constants
K_d_: Dissociationrate constants
K_D_: Affinities constants
RMSD: Root-mean-square deviation
ATU: Anti-thrombin units
FRE: Fibrinogen recognition exosite
